# Interdisciplinary Approaches to the Study of Listening Effort in Young Children with Cochlear Implants

**DOI:** 10.3390/audiolres12010001

**Published:** 2021-12-21

**Authors:** Amanda Saksida, Sara Ghiselli, Stefano Bembich, Alessandro Scorpecci, Sara Giannantonio, Alessandra Resca, Pasquale Marsella, Eva Orzan

**Affiliations:** 1 Institute for Maternal and Child Health—IRCCS “Burlo Garofolo”, 34100 Trieste, Italy; amanda.saksida@burlo.trieste.it (A.S.); stefano.bembich@burlo.trieste.it (S.B.); 2 “Guglielmo da Saliceto” Hospital of Piacenza, 29121 Piacenza, Italy; saraghiselli80@gmail.com; 3 Ospedale Pediatrico Bambino Gesù, 00165 Roma, Italy; alessandro.scorpecci@opbg.net (A.S.); sara.giannantonio@opbg.net (S.G.); alessandra.resca@opbg.net (A.R.); pasquale.marsella@opbg.net (P.M.)

**Keywords:** listening effort, listening fatigue, young children with cochlear implants, EEG, fNIRS, pupillometry

## Abstract

Very early bilateral implantation is thought to significantly reduce the attentional effort required to acquire spoken language, and consequently offer a profound improvement in quality of life. Despite the early intervention, however, auditory and communicative outcomes in children with cochlear implants remain poorer than in hearing children. The distorted auditory input via the cochlear implants requires more auditory attention resulting in increased listening effort and fatigue. Listening effort and fatigue may critically affect attention to speech, and in turn language processing, which may help to explain the variation in language and communication abilities. However, measuring attention to speech and listening effort is demanding in infants and very young children. Three objective techniques for measuring listening effort are presented in this paper that may address the challenges of testing very young and/or uncooperative children with cochlear implants: pupillometry, electroencephalography, and functional near-infrared spectroscopy. We review the studies of listening effort that used these techniques in paediatric populations with hearing loss, and discuss potential benefits of the systematic evaluation of listening effort in these populations.

## 1. Introduction

Early-life sensory inputs serve as fundamental building blocks for the functional organization of the developing brain. Rich auditory input, in particular, and later language development, have been identified as cornerstones of brain plasticity [1]. Even in the event of congenital hearing impairment, early detection and treatment of hearing loss are now possible thanks to the implementation of newborn hearing screening and early rehabilitation programs, providing an appropriate hearing input from the first months of life onward. In cases of profound hearing loss, cochlear implant (CI) surgery can be performed. Very early bilateral implantation is thought to significantly reduce the attentional effort required to acquire spoken language, and consequently offer a profound improvement in quality of life [2]. Despite the early intervention, however, auditory and communicative outcomes in children with CIs remain poorer than in hearing children [3]. The factors that contribute to such poorer outcomes are diverse and under the ongoing investigation [4]. Of these, one of the important issues appears to be fast automatic language processing developed early in life [5].

In CI users, the development of automatic language processing may be disrupted because of the degraded auditory input they are exposed to. Although sound processing has significantly improved in the recent years, the electrical signal transmitted through the CI devices nonetheless represents spectrally and temporally reduced auditory signal, compared to the signal received through the human ear [6,7,8]. The reduced spectral resolution and the elevated degree of spectral smearing cause interference in speech processing and recognition, especially in noisy environments (i.e., environments with background noises, with high reverberation, and with multiple interlocutors) [9]. Thus, CI users are typically slower for speech recognition in noise, even for speech that is accurately recognized [10].

Noisy environments compromise speech perception and comprehension also in populations with normal hearing [11]. Furthermore, listening in noisy environments requires more auditory attention, which elicits increased listening effort (the deliberate allocation of mental resources to overcome listening obstacles) and, subsequently, listening fatigue (exhaustion of mental resources devoted to a listening task) [12,13]. The age seems to play an important role in coping with background noise: while listening in noisy environments may be effortful but manageable for adults, it is more detrimental in children [14]. Preschool children with normal hearing are even more susceptible to background noise and tend to abandon the task of listening to speech at lower signal-to-noise ratios compared to older children [15].

Nonetheless, in comparison with normally-hearing populations, the effect of noise seems to be causing significantly more listening effort in individuals with hearing impairment, including the ones with CIs [16,17]. Again, listening effort seems to crucially depend on spectral resolution, which is degraded in the signal transmitted through the CI devices [18]. The differences between individuals with hearing impairment and normal hearing in perceived and objectively measured listening effort remain visible also in patients with bilateral CIs, although reduced in comparison with patients with unilateral CIs [19]. Despite the steep increase in the number of studies on listening effort in the last decade, very little is known about how young (preschool) children with (bilateral) CIs cope with noisy listening environments, compared to normally-hearing peers. Children with CIs pay less attention to speech, at least in the first years following implantation [20,21,22]. Although the exact relationship between listening effort, fatigue, attention to speech, and language processing is yet unclear, increased listening effort may critically affect attention to speech.

One of the reasons for the lack of studies on listening effort with very young children is of methodological nature. The majority of studies employing subjective measurements of perceived effort show that increasing environmental noise causes greater listening effort and fatigue [23,24]. Subjective measures, however, have significant drawbacks. They are more easily influenced by a number of environmental circumstances, making them less reliable in correlating with objective measurements. Furthermore, they are feasible in adults and school-age cooperative children, but not in younger children. Several objective psychoacoustic measures have been developed in parallel to assess listening effort, such as the use of dual-task paradigms, in which an auditory task (e.g., word/phrase repetition in noise) is administered together with a visual task (e.g., detection of visual targets) [19,25]. Again, administering these tests on young preschool children is frequently challenging, and their responses tend to be unreliable [10]. With recent technological advances, other objective measures have therefore been applied, such as assessing changes in heart rate and mean skin conductance [26,27,28], salivary cortisol levels for measuring fatigue [23], electroencephalography [29], functional near-infrared spectroscopy (fNIRS) [30], and pupillometry [31,32].

In the following paragraphs, we will present three objective physiological techniques that may prove to be applicable in the studies with very young (preschool) infants and children with CIs in the near future: fNIRS and electroencephalography in comparison with pupillometry, which has recently become the most widely used method for assessing listening effort. We discuss the advantages and drawbacks of each method, as well as the possibilities for their application in clinical practice.

## 2. Studies of Listening Effort Using Pupillometry

Human pupils respond with an involuntary contraction to light, near fixation (the change of focus), arousal, and cognitive activity (mental effort). The pupillary response induced by cognitive activity is closely linked to the activation of locus coeruleus (LC) neurons and the norepinephrine (NE) system. These structures (LC-NE) are involved in several processes including changes in stress, memory, and selective attention, along with general functions such as arousal and the sleep–wake cycle. Because of this close relationship, task-evoked pupillary dilation is used to assess the activity of the LC-NE system [33,34]. Measuring pupillary dilation as a response to arousal or cognitive activity is widely used in both clinical and psychological research. Currently, various eye-tracking devices allow for automatically obtaining information related to pupillary dilation with a good temporal resolution. As the pupil dilates involuntarily and automatically, pupillometry can be used for objectively measuring cognitive processes also in non-communicative subjects (e.g., animals, infants, uncooperative subjects). Using this methodology, several studies have measured cognitive responses to unexpected or novel events (the surprise effect or recognition), showing the sensitivity of pupillometry for unexpected auditory stimuli also in the paediatric population [35,36].

Several studies have used pupillometry to assess listening effort related to hearing abilities, sentence intelligibility, spectral resolution, lexical complexity, semantic context, and basic cognitive abilities in the adult population [12,17,18,37,38,39], with listening effort measurable even in conditions of good intelligibility [40], proving pupillometry to be a reliable tool for the assessment of listening effort [41,42]. Furthermore, a “threshold” level has been discovered in listening activities of increasing difficulty, beyond which the pupil stops dilating and abruptly reduces its diameter [34,43]. This phenomenon has been regarded as an effect of “disengagement” or withdrawal from the task, and hence as a signal of the onset of auditory fatigue [32].

In infants and children, pupillometry has been recently applied to a variety of auditory tasks. Infants’ and school-age children’s (4–10 years) pupils responded to infrequent/unexpected linguistic and non-linguistic stimuli [35,44,45]. Infants also showed pupil response to words segmented from a continuous speech stream [46]. Furthermore, pupillometry revealed monolingual and bilingual toddlers’ (24 months) sensitivity to mispronunciation of words [47,48]. Conversely, only a handful of studies have investigated listening effort in the paediatric population. In one recent work, the listening effort was assessed in school-aged hearing subjects, showing a relative effect of increased noise on the pupillary response [49]. In another study, binaural fusion abilities were observed in children with bilateral CIs. Binaural fusion may be compromised in children using bilateral CIs for a number of reasons, such as the use of devices implanted in different locations, the use of electrical stimulation to convey auditory information, and the degree and length of auditory deprivation. The study shows that binaural fusion is poor when inter-aural level cues are absent and further impaired when large asymmetries exist in the bilateral brain-stem pathways, leading to increased listening effort [50].

No research has been reported to date on the evaluation of listening effort with pupillometry in very young (preschool) children, either with normal hearing or hearing impairment, with no obvious reason for the lack of such studies, given that pupillometry has been successfully applied with infants and children in a variety of auditory tasks. Similarly, to our knowledge, no established protocol is available to this date, although clinical applications of pupillometry have been foreseen in several studies with patients with hearing loss [41,42,51,52,53].

## 3. Near-Infrared Spectroscopy (NIRS) of the Brain in Audiology

The near-infrared spectroscopy (NIRS) technique detects changes in oxygenated haemoglobin (HbO2) and deoxygenated haemoglobin (Hbb) in biological tissue, using the fact that haemoglobins absorb light at the wavelengths between 650 nm and 1000 nm [54]. As biological tissue is transparent to near-infrared light, and light photons can pass through tissue at these wavelengths, haemoglobins can be detected by measuring the photons that remain unabsorbed and re-emerge from the somatic surface. Furthermore, because HbO2 and Hbb have absorption peaks at different levels of the infrared light spectrum, both can be detected using at least two distinct wavelengths (830 nm and 690 nm, respectively). The attenuation of the light signal re-emerging from the examined tissues may then be utilized to infer variations in the concentration of the two types of haemoglobin associated with blood flow. Among the primary scientific and clinical applications of this method is the evaluation of changes in HbO2 and Hbb concentrations that occur in specific regions of the cerebral cortex over a specific time frame. The change in HbO2 concentration is considered to be an indicator of cerebral blood flow and, as a result, the functional activity of a specific cortical region. Instead, the shift in Hbb concentration is seen as an indicator of oxygen metabolism [55].

Thanks to the non-invasiveness, the absence of harmful radiation, or the need for sedation, good tolerance to movements of the patient, the portability of the equipment, and the relatively good temporal resolution, this technique is particularly suitable for studying the physiology of the cerebral cortex both in infants and very young children, who are not suitable for other functional neuroimaging methods, such as functional magnetic resonance imaging (fMRI) or positron emission tomography, due to obvious reasons inherent to the procedures. Nevertheless, despite its great temporal resolution, multichannel NIRS has only a few centimetres of spatial resolution and cannot yet be matched to the precision of fMRI, which has a discriminative capability of 1 mm. It is also worth noting that NIRS only reaches depths of around 3 cm, limiting functional cortical activity imaging to the most superficial regions [56,57].

As a functional neuroimaging technique unaffected by electrical artifacts, NIRS has been found to be particularly compatible with the presence of the CIs. In recent years, it has been used to examine functional activation of the auditory cortex, in particular the lateral temporal lobe and superior temporal sulcus, in both hearing-impaired adults and children with CIs [30]. Patients with a CI and good speech perception were found to have a temporal cortical activation similar to normal-hearing people, while implanted patients presenting a difficult perception of language stimulation had less activation of the same portion of cortex [58]. However, recent findings point to the possibility that cortical reorganization, as experienced by CI users, may enhance speech perception and processing thus representing a compensation mechanism [59]. Multichannel brain NIRS has also been used to investigate the neuroplastic reorganization of auditory cortices in profound-deaf subjects without a CI, using visual and vibro-tactile stimuli [60]. It was observed that visual stimuli, but not tactile stimuli, were able to activate the temporal cortex. In light of these findings, the use of brain NIRS as a functional neuroimaging tool has been recommended to guide the programming of post-implant rehabilitation intervention, according to the improvement of the patient’s auditory and linguistic outcomes. In addition, assessment of cortical reorganization occurring after a prolonged auditory deprivation in potential CI recipients could help predict possible functional outcomes of the intervention.

On the other hand, fNIRS has been only recently employed for evaluating cognitive load. Research has focused on the adult population and examined the effects of attentional burden on the frontal cortex, showing an inversely proportional relationship between HbO2 and the amount of cognitive load, proving HbO2 to be an indicator of cognitive load during working memory and control, as well as visuomotor tasks [61,62,63]. HbO2 concentrations in prefrontal cortex were also positively associated with listening effort in older adults with hearing aids [64]. New results from a study with normally hearing adults indicate that listening effort is partly reliant on higher cognitive auditory attention and working memory mechanisms in the frontal lobe and on hierarchical linguistic computation in the brain’s left hemisphere [65]. In addition, functional connectivity was assessed using fNIRS, with higher cross-modal connectivity for auditory stimuli associated with better speech recognition abilities, pointing to a new pattern of functional reorganization that is related to successful hearing restoration with CIs [66].

While fNIRS has not yet been used as a method to assess listening effort in children, it has been extensively used in the last decades to show auditory attention, attention to speech, and speech processing in infants [67,68,69,70] and children [56,57]. Therefore, while acknowledging its limitations, brain NIRS can be proposed for the study of listening effort also in paediatric population with CIs. Similarly, although the measures of speech processing with NIRS have been proposed for clinical practice, especially in the area of language impairment and speech processing in schizophrenia [71,72], clinical protocols for the usage of NIRS to assess speech processing in children with CIs are yet to be established.

## 4. Studies of Listening Effort by EEG

The Electroencephalogram (EEG) is a long-used method for studying brain activity, able of detecting the synchronous excitatory or inhibitory activity of large groups of pyramidal neurons, whose major axis is perpendicular to the cortical surface. Such activity is traditionally classified into beta (>13 Hz), alpha (8–13 Hz), theta (4–8 Hz), and delta (0.5–4 Hz) frequencies. Unlike functional MRI, which has a high spatial resolution, EEG has a high temporal resolution, allowing it to detect changes in brain activity within fractions of a second. Because of this characteristic, as well as its non-invasiveness and low cost, EEG looks to be a very promising tool in the investigation of objective neural correlates of effort and cognitive engagement during listening. An increase in alpha-band oscillatory activity in adult subjects is positively correlated with cognitive effort and attention sustained during listening, attributing this phenomenon to the synchronous activity of neural networks responsible for selective inhibition processes [73]. This finding has been further confirmed by a recent study using combined EEG (alpha activity) and pupillometry measurement during speech processing in noise, which has revealed that both methods are sensitive to changes in spectral resolution [74].

The most significant disadvantage of EEG is its low spatial resolution, which makes determining the cerebral source of recorded activity challenging, even if advances in technological developments are leading to increasingly improved spatial resolution, allowing for more accurate reconstruction of functional maps of the cerebral cortex during listening tasks. Furthermore, the EEG is typically “immature” in the paediatric age group, making it difficult to interpret the oscillations during task execution using adult-derived criteria. A clinical study on paediatric patients with bilateral sensorineural hearing loss found an increase in EEG alpha activity during a listening task in noise vs. a listening task in quiet settings, which was mostly mapped in the parietal cortex [29]. This suggests the activation of neural pathways that exert an inhibitory action against stimuli “irrelevant to the task,” such as competing noise. However, the localization of the cortical source of alpha activity is not direct, but rather derived by a statistical estimate. Therefore, these results should be taken with caution.

One possible way to overcome the poor spatial resolution of EEG was recently found in combining NIRS and EEG techniques [75,76,77,78]. Although the combination has not been used to study listening effort, simultaneous NIRS-EEG registration of brain response may not only improve spatial resolution of the EEG signal, but also provide a better understanding of the mechanisms involved in cerebral activation, and help to avoid misleading interpretation of NIRS [79].

A recent study [80] used high spatial resolution EEG during a listening-in-noise task to compare a group of normally-hearing children to a group of children with single-sided deafness (SSD). When compared to normally-hearing participants, who lateralised all EEG activity in the left hemisphere, children with SSD revealed less significant lateralisation of the activity, which was likely due to more diffuse cortical activation as a result of recruitment of additional regions. Given this conclusion, using a larger cortical area to perform the listening task represents a promising objective measure of the listening effort. As expected, the same study showed that normally-hearing children always lateralise EEG activity to the left, whereas children with SSD tend to lateralise in the hemisphere facing the noise source. This incomplete specialization of the cerebral hemispheres probably reflects the plastic modifications of cortical networks that occur after prolonged mono-aural auditory deprivation.

To conclude, EEG can allow objective measurement of auditory strain in paediatric age by: (1) recording of task-related phasic oscillations of specific activities known to be associated with cognitive engagement and localized in specific cortical regions (e.g., increase of alpha activity in the parietal cortex); (2) examining the extent of lateralisation of cortical activity based on the theory that poor lateralisation corresponds to the recruitment of a larger cortical area and therefore a greater investment of cognitive resource; and (3) investigating the manner of lateralisation of cortical activity: the more this deviates from the norm, the less hemispheric specialization and, as a consequence, the more cognitive energy expended by the cortex during a listening task. It is, however, to be noted that, similarly to the previously described techniques, EEG has not been established as a clinically approved technique for measuring speech processing and listening effort in children with cochlear implants, although EEG correlates of listening effort have been recently proposed as a clinical measure of the exerted effort during various hearing aid configurations in adults [81].

## 5. Conclusions

Given the current state of the research, and strengths and weaknesses of each of the methods described, none of them is likely to be sufficient for a comprehensive and exhaustive evaluation of listening effort by itself. However, the presented techniques offer a window of opportunity to understand the challenges and limits of attention to speech in young children with Cis, who are generally challenging to test with behavioural measures. In line with recent publications on each of the presented methods [51,52,53,72,81], we believe that a battery of clinical tests that would include an objective physiological assessment would be desirable. Accordingly, it is essential that more studies be undertaken to validate these measures. The availability of a test battery for clinical use capable of consistently and precisely assessing listening effort and fatigue would allow significant advancement in terms of the indication for treatment of deafness, and the evaluation of the therapy’s outcomes. Indeed, they would allow the traditional assessment of the outcome in terms of impairment to be combined with the assessment of disability caused by deafness.

## Data Availability

The study did not report any data.
